# The compound event that triggered the destructive fires of October 2017 in Portugal

**DOI:** 10.1016/j.isci.2023.106141

**Published:** 2023-02-04

**Authors:** Alexandre M. Ramos, Ana Russo, Carlos C. DaCamara, Silvia Nunes, Pedro Sousa, P.M.M. Soares, Miguel M. Lima, Alexandra Hurduc, Ricardo M. Trigo

**Affiliations:** 1 Institute of Meteorology and Climate Research, Karlsruhe Institute of Technology, Hermann-von-Helmholtz-Platz 1, Building 435, Eggenstein-Leopoldshafen, 76344, Karlsruhe, Germany; 2 Faculdade de Ciências, Universidade de Lisboa, Instituto Dom Luiz, 1749-016 Lisbon, Portugal; 3 Instituto Português do Mar e da Atmosfera (IPMA), 1749-077 Lisbon, Portugal; 4 Departamento de Meteorologia, Universidade Federal do Rio de Janeiro, Rio de Janeiro 21941-919, Brazil

**Keywords:** earth sciences, Atmospheric science, Climatology

## Abstract

Portugal is regularly affected by destructive wildfires that have severe social, economic, and ecological impacts. The total burnt area in 2017 (∼540,000 ha) marked the all-time record value since 1980 with a tragic toll of 114 fatalities that occurred in June and October events. The local insurance sector declared it was the costliest natural disaster in Portugal with payouts exceeding USD295 million. Here, the 2017 October event, responsible for more than 200,000 ha of burnt area and 50 fatalities is analyzed from a compound perspective. A prolonged drought led to preconditioned cumulative hydric stress of vegetation in October 2017. In addition, on 15 October 2017, two other major drivers played a critical role: 1) the passage of hurricane Ophelia off the Coast of Portugal, responsible for exceptional meteorological conditions and 2) the human agent, responsible for an extremely elevated number of negligent ignitions. This disastrous combination of natural and anthropogenic drivers led to the uncontrolled wildfires observed on 15 October.

## Highlights

• October 2017 wildfires burned 200,000 ha and 50 fatalities in Portugal
• Preconditioned cumulative hydric stress of vegetation due to long-term drought
• Hurricane Ophelia responsible for exceptional meteorological conditions
• Compound event nature of the October 2017 wildfires

## Graphical abstract

**Figure:**
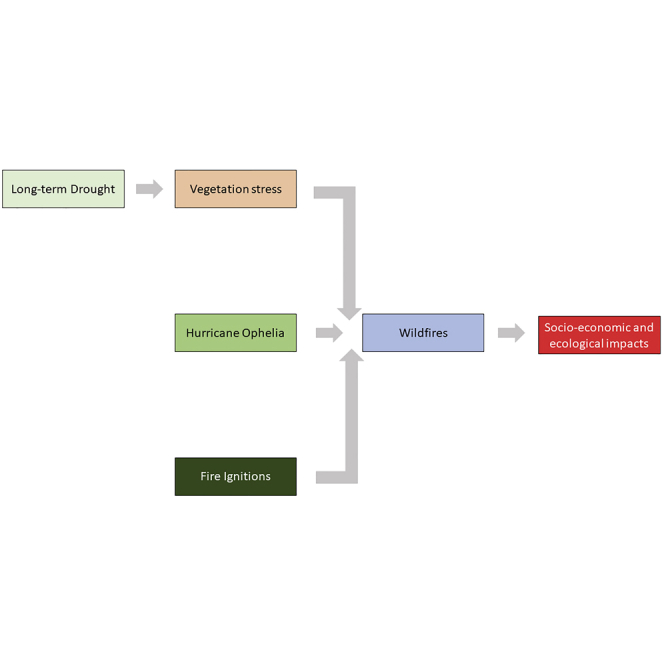

## Introduction

The climate conditions of the western Mediterranean are characterized by the coexistence of sub-tropical and mid-latitude influences.^1^ The dependence of Mediterranean ecosystems in terms of precipitation amounts and soil moisture makes these biomes particularly sensitive to weather-driven natural hazards, namely droughts,^2^^,^^3^^,^^4^^,^^5^^,^^6^ heatwaves,^7^^,^^8^^,^^9^^,^^10^^,^^11^ wildfires,^12^^,^^13^^,^^14^ and floods.^15^^,^^16^^,^^17^

Recently, several authors have stressed that some of the most extreme weather-driven natural hazard events result from a combination of extremes, in what has become known as compound events.^18^^,^^19^^,^^20^ Ultimately, the interaction between different physical processes at multiple temporal and spatial scales can lead to the exacerbation of the impacts compared to when hazards occur individually.^20^^,^^21^^,^^22^ This interplay between different co-occurring drivers or cascading events have been widely reported in recent years, namely focusing on soil moisture-temperature feedbacks,^23^^,^^24^^,^^25^ on the relation between dry and hot extremes,^26^^,^^27^^,^^28^^,^^29^ as well as between weather and fires,^19^^,^^30^ and on the impacts on different sectoral activities.^31^ In this context, it is crucial to analyze this interplay following a compound event approach in order to foster a deeper understanding on how the different hazards interact, and also on how such compound events can induce more extreme impacts when compared to the occurrence of an isolated extreme event. This type of approach also has the advantage of allowing for the development of more robust evaluation and mitigation measures when facing the occurrence of such extremes.^22^

Recently, a new framework for studying compound events was proposed by Zscheischler et al.^22^ According to these authors a compound weather event refers to a combination of drivers and/or hazards that contribute to societal or environmental risks. This type of framework can be particularly relevant within the scope of climate change as, under the projected warming and drier conditions,^32^ the occurrence of more extreme compound events is expected to be enhanced,^33^ namely over semi-arid susceptible areas such as the Mediterranean.^29^^,^^34^

In the last two decades, southern Europe was struck by several major compound events (e.g. summer heat waves and droughts), some of them with heavy socio-economic impacts, especially on food production, public health, air pollution, and mortality.^35^^,^^36^^,^^37^^,^^38^^,^^39^^,^^40^^,^^41^^,^^42^^,^^43^ Some of these events led to catastrophic fire seasons, such as those in 2005^44^ and 2017^14^ in Portugal, or the one in 2007 in Greece.^19^ The occurrence of catastrophic wildfire events due to compound drought and heat extremes are not limited to Europe, but also takes place in other regions of the world such as Brazil,^45^ the US,^46^ and Australia.^47^ In addition, some studies focus also on the boreal regions.^48^^,^^49^

Portugal is regularly affected by large and destructive wildfires leading to serious impacts at social, economic, and ecological levels.^14^^,^^40^^,^^42^^,^^50^ Since 1980, the mean annual burnt area has been around 115,000 ha with large inter-annual variability, including particularly severe years, such as 2003 (∼425,000 ha), 2005 (∼350,000 ha), or the all-time record value of 2017 (∼540,000 ha).

Whatever the perspective, the 2017 fire season in Portugal was outstanding, recording the highest total burned area (∼540,000 ha) ever registered since reliable measurements started in 1980, representing nearly 60% of the total burn area in Europe in that year. More significantly, this exceptional fire season set the tragic toll of 114 fatalities.^14^^,^^51^^,^^52^ Furthermore, the unusual extent of the 2017 fire season implied that the two most tragic events occurred prior (17–20 June) and after (15–17 October) to the official fire season window set by the Portuguese authorities, which until that year, encompassed only the months of July, August, and September. The economic losses due to the 2017 wildfires in Portugal totaled almost USD1.2 billion, and the local insurance sector declared the costliest natural disaster in the country’s history with payouts exceeding USD295 million.^53^

The different nature of the June and October events is worth being emphasized. It has been shown that the June episode was triggered by a record-breaking heat wave week in mid-June that affected western Europe and, in particular, the entire Iberian Peninsula.^38^ The case of October was shaped by the coupling of very dry vegetation with very strong southerly winds.^54^ Vegetation stress was due to a persistent drought situation,^55^ whereas the southerly winds were steered by the close offshore passage of hurricane Ophelia moving northward.^52^ It is worth noting that the socio-economic impacts of the Hurricane Ophelia were not only felt in Portugal but struck Ireland in its extratropical transition having caused major damages, losses of around 90 million euros^56^ and 3 casualties.

Several studies and official reports analyzed the 2017 fire season,^42^^,^^52^^,^^54^^,^^57^ but they lack the holistic perspective of analyzing the compound nature of the event. Thus, the main goals of this paper are 2-fold:

• to show the compound nature of the exceptional fires that occurred in Portugal in October 2017 by assessing the different drivers and hazards that contributed to it;
• to characterize in detail the different components of the fire weather index (FWI) from a bivariate perspective (concurrent versus accumulated effects of meteorological conditions), with the aim of objectively establishing their different contribution to the extreme value of FWI, and to the exceptional fire events of October 15.

## Results

### The 2017 fire season

**Figure 1:**
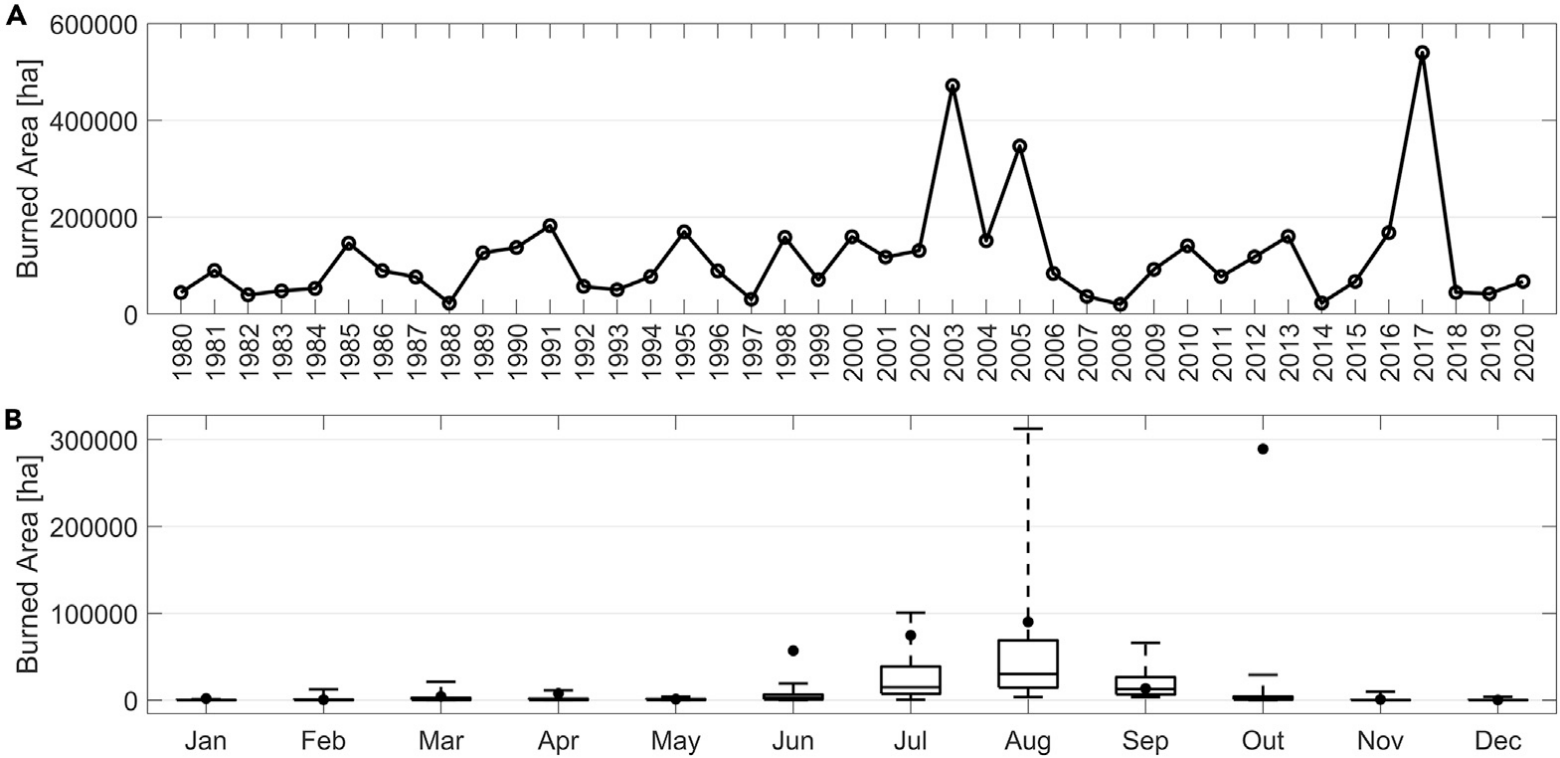
Inter-annual and intra-annual variability of burned area (ha) in Portugal (A) The inter-annual variability of burned area (ha) in Portugal for the period 1980–2020. (B) Annual cycle of monthly burned areas in Portugal. For each month and for the period 1980–2020 (excluding 2017), boxes delimit the first and third quartiles, the segment inside the box indicates the median and the whiskers extend down from the minimum up to the maximum. The annual cycle of 2017 is represented by the black circles, and it is worth noting the exceptionality of the area burned in October.

Burned area in Portugal presents a marked inter-annual variability (Figure 1A), with the three largest peaks, in 2003, 2005, and 2017, having occurred in the second half of the period analyzed (1980–2020), which starts when fire events have begun to be officially recorded. The inter-annual variability of burned area in Portugal is partly attributable to thermal and hydric stress conditions of vegetation as a result of the amount of precipitation in the fire season and in the preceding late spring season, and partly to the occurrence of atmospheric circulation patterns in the summer that induce extremely hot and dry spells over western Iberia.^58^ The increasing occurrence of drought episodes and heatwaves further enhance the onset of extreme episodes and are related to global warming.^27^^,^^59^ Striking examples of extreme years are 2003, that was characterized by exceptionally warm weather in Europe during the first two weeks of August, when a devastating sequence of large fires was observed,^40^ and 2005 when the Iberian Peninsula was hit by the worst drought episode recorded since the beginning of the 20^th^ century.^36^

The record-breaking fire season in Portugal took place in 2017, when the total burn area reached the amount of 540,000 ha, but the 2017 fire season is also outstanding for its highly unusual annual cycle (Figure 1B), the months of June and October both presenting the highest monthly amounts recorded in 1980–2020. The amount recorded in October is especially impressive since it almost reached the maximum of burned area in August and represents by far the largest contribution to the annual amount. This is worth being noted since June and October are both out of the traditionally defined fire season spanning from July to September. However, it is noteworthy that the monthly amounts of burned area in June and October 2017 were well above the maximum of the respective month (Figure 1B) when considering the period 1980–2020 (2017 being excluded).

**Figure 2:**
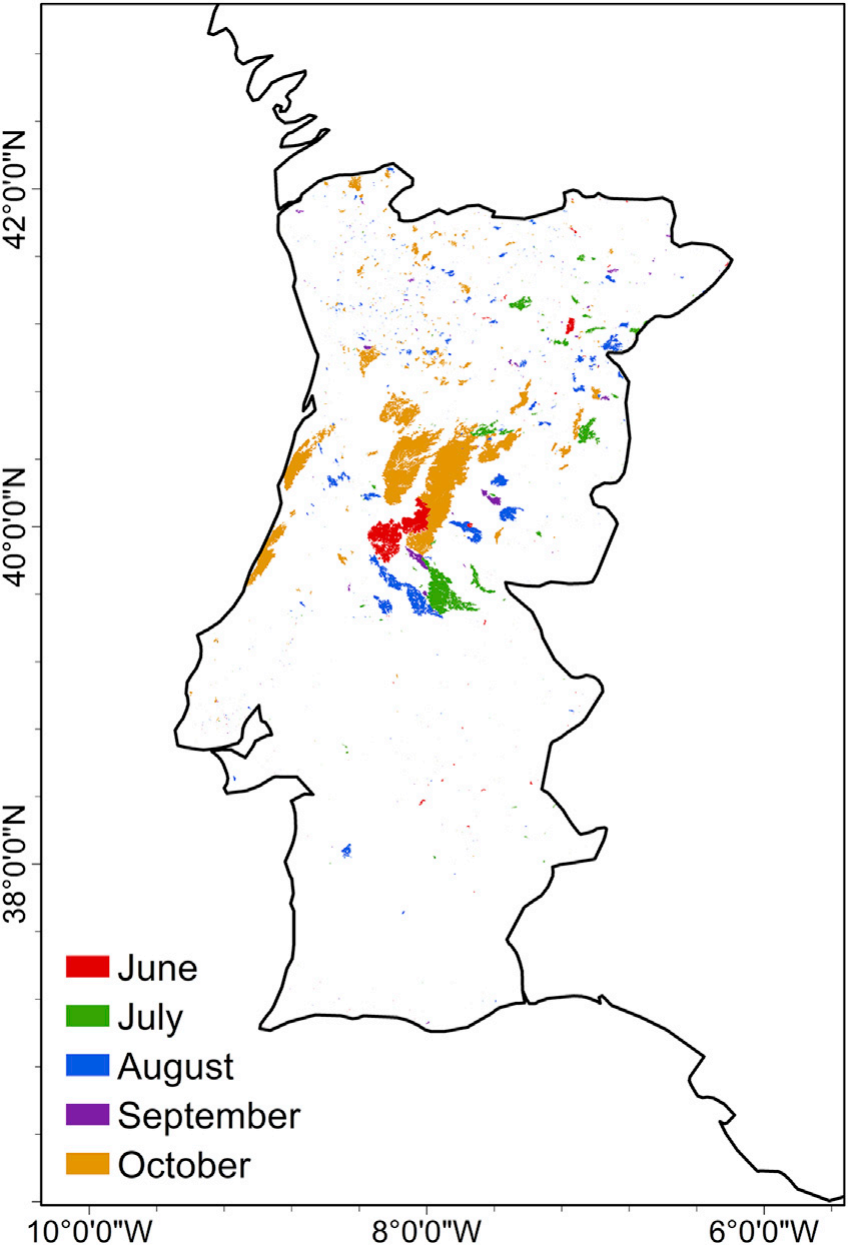
Location of burned areas resulting from fires that took place between June and October 2017 The burned areas are colored according to the month of occurrence.

The exceptional monthly values of June and October 2017 were due to two extremely large fire events that took place on 17–20 June and 15–16 October (Figure 2), respectively. In the June event, the largest fires occurred in central Portugal (Pedrogão-Grande and Gois, red areas in Figure 2), the burned area reaching circa 50,000 ha and the fast convective nature of the Pedrogão fire contributing to the 64 fatalities registered.^52^^,^^60^ The October event was responsible for more than 200,000 ha of burnt area and 50 fatalities in Portugal. In the latter, more than 500 ignitions were recorded and several of them developed into major fires burning a very large area in a very short period of less than 10 h. The maximum rates of spread were above 3 km/h and up to 9 km/h,^52^ and an average of 10,000 ha burned per hour was recorded in central Portugal.^57^ From this point onwards, we will focus on the October event paying special attention to the preceding drought conditions and the importance of the passage of hurricane Ophelia off the coast of Portugal.

### The 2016/2017 drought and effect on the vegetation stress

The 2016/2017 drought was unprecedented and affected western and central Europe.^55^ The main difference from other European droughts is that this event displayed a highly unusual spatial pattern affecting both northern and southern European regions, with drought conditions affecting more than 90% of central-western Europe, hitting record-breaking values (regarding the historical period of 1979–2017) in 25% of the area. The 2016–2017 drought was also outstanding in terms of persistence, with the accumulated values for most months of the period laying below the 10th percentile of the climatological distribution, reaching far lower values than the 2004–2005 drought at the European level.^55^ The main dynamical driver of the drought was the consecutive occurrence of blocking anticyclones and sub-tropical ridges, sometimes displaced from their typical locations.^61^ This led to latitudinal shifts of the jet stream and record-breaking positive geopotential height anomalies over most of the continent. The extreme drought conditions had different socio-economic impacts such as crop and water supply failures, forest fires, and hydroelectric power production reduction. In addition, the reduced soil moisture in Europe in late spring 2017 contributed to several heat waves^39^^,^^55^^,^^62^ that occurred in the summer 2017.^38^^,^^63^

**Figure 3:**
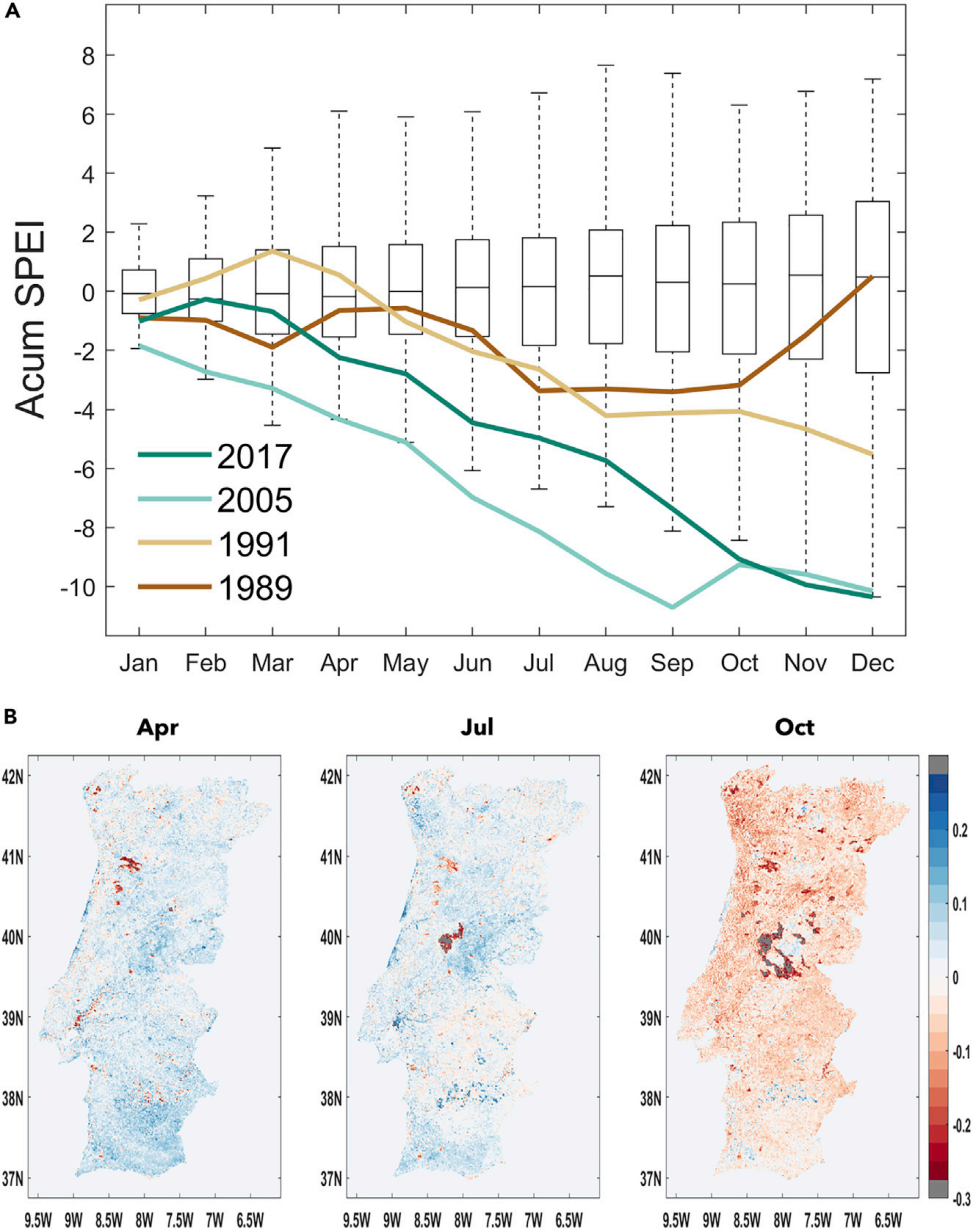
2017 accumulated monthly SPEI-1 and NDVI anomalies Accumulated monthly SPEI-1 from January 2017 to December 2017 (A) and NDVI anomalies for the months of April (Apr), July (Jul), and October (Oct) 2017 (B). The lines in (A) represent the most outstanding drought years in Portugal.

Regarding the Iberian Peninsula, dry conditions started in July 2016 (as indicated by the 12-month SPEI, supplemental information Figure S1) and lasted until May 2018, indicating that this drought event can be categorized as a hydrological drought persisting for more than a year. While the drought severity varied in space and time, snapshots of the 12-month SPEI reveal widespread dry conditions for all months of the analyzed period. In order to analyze the influence of the drought conditions during the 2017 fires, we have represented the evolution of the accumulated 1-month SPEI averaged over continental Portugal, as displayed in Figure 3A, for the monthly values of some of the driest years in southern Europe. Accumulated 1-month SPEI values, for Portugal, were below −10 at the end of the period of study, marking the exceptionality of the event which was considered the most severe European drought at the continental scale, at least since 1979.^55^

The impact of drought conditions on vegetation hydric stress is assessed by means of the Normalized Difference Vegetation Index (NDVI), here shown for April 2017, July 2017, and October 2017 (Figure 3B). Pixels in gray represent those that are contaminated by clouds and therefore not considered in the remaining analysis. During the hydrological year of 2017 negative anomalies of NDVI were particularly spread throughout the country from August to October, whereas from May to July they were mainly confined to the southern region. Therefore, the previous months of the fires show clear negative values of NDVI anomalies throughout the country.

### The role of Hurricane Ophelia

**Figure 4:**
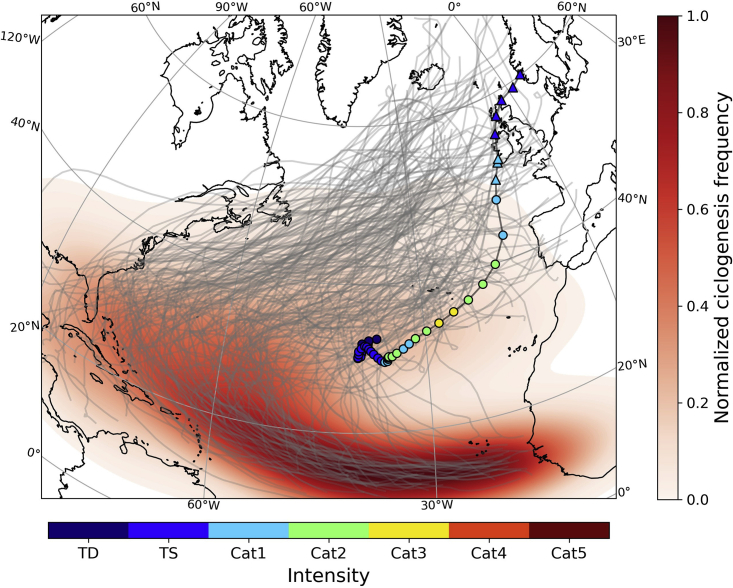
Tracks (gray lines) and ciclogenesis density (color shaded) of TCs that reached the Northeastern section of the North Atlantic basin (North of 25°N, East of 40°W) for the 1851–2020 time period Emphasis on Ophelia (2017) that shows its track (colored circles) as well as each 6-h location and intensity.

It is now widely recognized that, although not landing on the Iberian Peninsula, hurricane Ophelia caused widespread impacts on the region, being one of the catalyzers of the October 2017 Iberian wildfires.^54^^,^^57^ Moreover, hurricane Ophelia fits within the context of an apparent increase in tropical cyclone activity in the north-eastern part of the North Atlantic basin,^64^^,^^65^ including four recent events in the north-eastern part of the North Atlantic basin all producing impacts in either continental Portugal (Ophelia in 2017, Leslie in 2018, and Alpha in 2020) or in the archipelago of Azores (Lorenzo in 2019). Hurricane Ophelia in October 2017^66^ and Hurricane Leslie in 2018^67^ affected the Iberian Peninsula with important socio-economic impacts. As mentioned previously, Ophelia track was off the coast of mainland Portugal, as Category 1 hurricane, being one the few tropical cyclones that affected the Iberian Peninsula since 1851 (Figure 4). Hurricane Ophelia had its origin in the first week of October, farther north of typical cyclogenesis areas for most TCs in the NA basin,^65^ where it stayed relatively stationary for about two days, gathering intensity over the warm waters, due to the presence of a mid-latitude ridge located north. On October 12th, the ridge erosion helped to induce the gradual movement of Ophelia eastward while it intensified into its maximum intensity of 100 kt (south of Azores), corresponding to a category 3 on the Saffir-Simpson hurricane wind scale.

**Figure 5:**
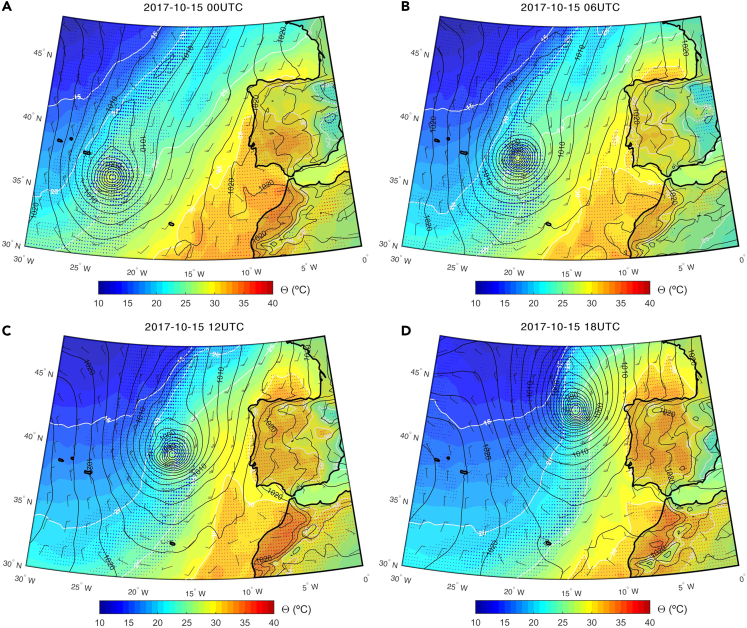
Evolution of the sea level pressure (black contours), potential temperature (colored shading), and relative humidity (blue/red dots) in the lower troposphere (1000-700 hPa), as well as surface wind (wind barbs) at the onset of the October 2017 wildfires in Portugal (October 15, 2017), with hurricane Ophelia located offshore The hours correspond to (A) 00UTC; (B) 06 UTC; (C) 12UTC and (D) 18UTC. The increasing size of the blue (red) dots depicts relative humidity values higher than 70%, 80%, and 90% (lower than 30%, 20%, and 10%) of the climatolgy, respectively.

As Ophelia was located approximately mid-way between the Azores and continental Portugal (October 15), and still classified as a hurricane, despite the unusual location for such a strong tropical cyclone, a very strong southerly flow was steered over continental Portugal. This intense meridional circulation due to the strong pressure gradient associated with Ophelia is depicted in Figure 5. This synoptic configuration produced a very strong advection of warm and dry air originating from northern Africa, as visible by the high potential temperature (Θ) values over western Iberia during that day. High Θ values provide a typical fingerprint of desertic air masses intrusions, which are recurrent over Iberia,^62^ and are often responsible for a significant fraction of extreme temperature events in the region.

On 15 October, hurricane Ophelia was sufficiently close to the Portuguese coastal region to foster the transport of a warm and dry air mass, but it was still located too far from continental Portugal to put the territory under the direct influence of the moist tropical air associated with the cyclones’ core and outer bands, which could provide some relief from severe fire weather conditions. This is clearly observed as the day progressed, and wildfires started, with a very steep west-east gradient for both Θ and relative humidity (Figure 5 panel c-d).

On 15 October, local meteorological conditions including sustained southerly wind speeds between 30 and 40 km/h and maximum wind speed between 50 and 80 km/h were recorded in central Portugal (where most fires occurred) during the afternoon. As a consequence of the previously described advection mechanism, on 15 October near-surface relative humidity below 20% was widespread across Portugal^54^ and 70% of the weather stations recorded values above 30 °C, an unusually high value for October in Portugal. In fact, most of the coastal weather stations located north of Lisbon broke their October maximum temperature record during this day.^68^ During the following day (16 October, supplemental information Figure S2), Ophelia moved northward in the direction of Ireland, leading to the intrusion of cold and humid air occurred leading to rainfall in western Iberia, heavily assisting the extinction of wildfires.^51^

In short, the preconditioning record-breaking drought that had been affecting western and central Europe since July 2016^55^ and its effects on vegetation stress in Portugal played a major role as discussed previously. The unusual presence of hurricane Ophelia, as well as its very specific positioning, were determinant to produce the exceptional meteorological conditions observed at the date of the fires’ onset (i.e. very high temperatures, very low relative humidity, and strong surface wind).

In the next section, we will discuss how the preconditioned 2016/2017 drought and the exceptional meteorological conditions on October 15 affected the likelihood of an extreme fire event from a compound event perspective.

### The extreme fire weather index on 15 October, the compound perspective of the event

The exceptionality of the 15 October 2017 event is evident when comparing the components of the Canadian Forest Fire Weather Index System (CFFWIS) for that day with the respective components for the 41-year period 1980–2020. The CFFWIS rationale and its integrated components are fully described in the STAR Methods section. The CFFWIS includes the Fine Fuel Moisture Code (FFMC), the Duff Moisture Code (DMC) the Drought Code (DC), the Initial Spread Index (ISI), the Build Up Index (BUI), and the Fire Weather Index (FWI).

**Figure 6:**
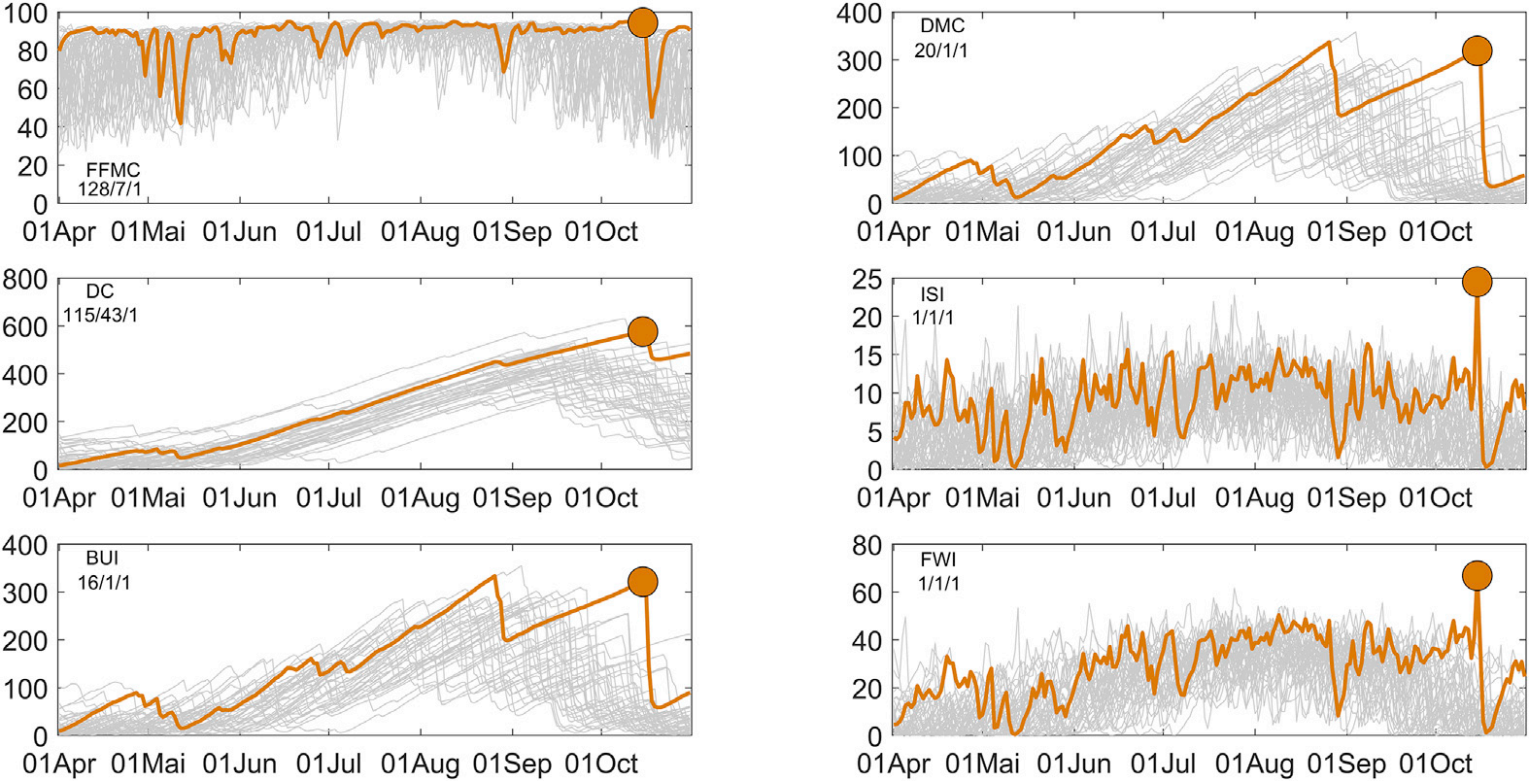
Time series of daily values of the six components of CFFWIS (as identified by the labels in each panel) for the period from 1 April to 31 October 1980–2020 Time series for 2017 is colored in orange and those for the remaining years are colored in gray. The three integers separated by slashes in top left corner in each panel indicate, for the respective component, the ranking of the value on 15 October 2017 (identified as a solid orange circle) among the values recorded in 1980– 2020, respectively for the period between 1 April-31 October (8774 events), for the month of October (1271 events) and for the 15^th^ October (41 events).

Results are presented in Figure 6 and it is worth noting that values of all components of CFFWIS (i.e., FFMC, DMC, DC, BUI, ISI, and FWI) on 15 October 2017 rank first among the 41 daily values of respective components observed on the same day of the year for the period 1980–2020. More significantly, when comparing the components on 15 October 2017 with the respective components in October 1980–2020 (31 × 41 = 1271 daily values), all components rank first, except FFMC and DC that rank 7 and 43, respectively. When the comparison extends to the period 1 April-31 October 1980–2020 (214 × 41 = 8774 daily values), ISI and FWI rank first, BUI ranks 16, DMC ranks 20, DC ranks 115 and FFMC ranks 128. It is worth stressing that the slow-varying nature of drought-related indices (DC, DMC, BUI) implies that the slightly top rank attained by 15 October in this last all-year comparison masks the fact that in only one of the previous years, the 15 October 2017 value was surpassed several times.

The high ranks of FFMC, DMC, and DC on 15 October 2017 clearly indicate that both short-term and long-term atmospheric conditions contributed to the exceptionally high stress of the vegetation, both in terms of proneness to ignition and to fire spread that translate into the high ranks of ISI and BUI. It is also worth stressing the role played by the passage of hurricane Ophelia in steering extremely high wind speed, which contributed to the top ranks of ISI and FWI on 15 October 2017.

**Figure 7:**
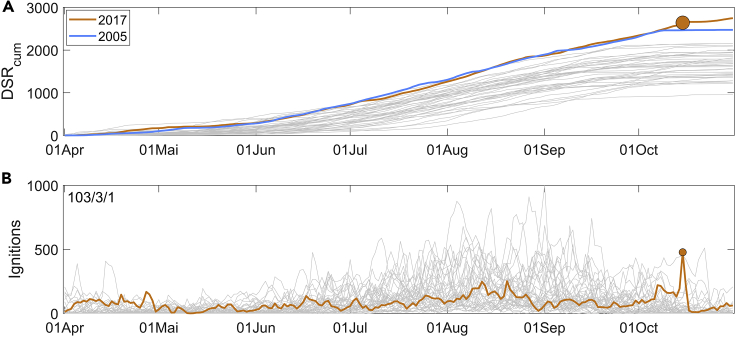
Time series of daily values of DSR_cum_ and Ignitions for the period from 1 April to 31 October 1980–2020 Time series of daily values of DSR_cum_ for the period from 1 April to 31 October 1980–2020 (A) and (B) as in Figure 6, but for the time series of the ignitions. Time series for 2017 and 2005 are colored in orange and blue, respectively, and those for the remaining years are colored in gray. The solid orange circle identifies the value of DSR_cum_ on 15 October 2017.

The exceptional level of cumulative stress on vegetation that was induced by long-term atmospheric conditions during the spring and summer 2017 is conveniently assessed when comparing the time series of cumulative Daily Severity Rating (DSR_cum_) with the corresponding curves of the period 1980–2020 (Figure 7A). The Daily Severity Rating (DSR) is an extension of CFFWIS and is directly obtained from FWI, and rates the difficulty of controlling fires.^69^ As a rating of vegetation stress, we will make use of cumulative DSR (DSR_cum_) that, for each day of the year, is simply defined as the accumulated daily values of DSR since April 1 of that year (see the STAR Methods section for more detail).

From 13 August onwards, the curves of DSR_cum_ for 2017 and 2005 assume a leading position, 2005 ranking first until 9 September, and 2017 taking the first position after this date and reaching maximum values in the last three weeks of October. As already mentioned, the Iberian Peninsula was affected in 2005 by the most intense drought episode ever recorded^36^ and, in terms of cumulated vegetation stress, 2017 closely follows 2005 and even surpasses it in October.

**Figure 8:**
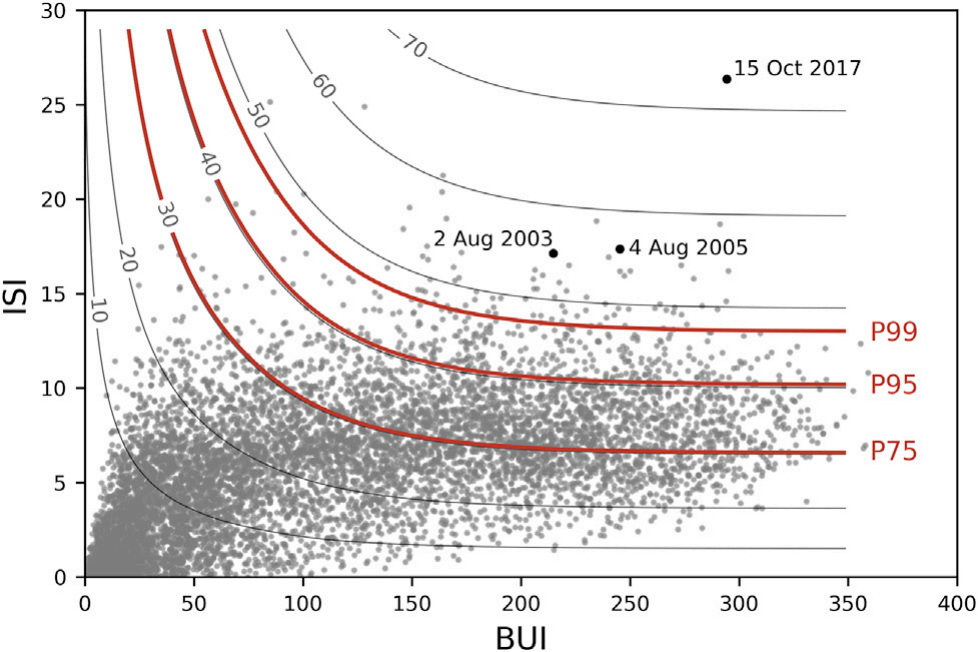
FWI as a function of BUI and ISI Values for the period 1 April-31 October 1980–2020 are represented as gray circles, and the values observed on 2 August 2003, 4 August 2005, and 15 October 2017 are highlighted as black circles and labeled. Orange curves represent the contour lines of percentiles 75, 95, and 99 of the set of observed values of FWI. The grey curves correspond to diferent FWI isolines between 10 and 70.

A comparative assessment of the roles played by long-term and short-term atmospheric conditions may be performed by analyzing the relative contributions of BUI and ISI to FWI. Figure 8 shows FWI as a function of BUI and ISI, and the relative contributions of these two components reflect on the slope of contour lines of FWI. For BUI below 75, contour lines present a very high slope, indicating that ISI has a very small contribution; on the contrary, for values of BUI above 175, contour lines are almost horizontal, and therefore ISI has a prominent contribution to FWI. Figure 8 also presents (gray circles) the values of BUI and ISI observed during the period 1 April-31 October 1980–2020. The outstanding values of BUI, ISI, and FWI on 15 October 2017 are conspicuous, and the contribution of ISI to FWI is worth stressing. For comparison purposes, two other outstanding days are also represented, namely 2 August 2003 and 4 August 2005 associated with daily values of area burned of 97,509 ha and 25,291 ha, respectively, well below the recorded value of 252,734 ha that was recorded on 15 October 2017. Values of FWI on 2 August 2003 and 4 August 2005 are above percentile 99 (but well below the value on 15 October 2017), and the contribution of ISI is also worth noting in both cases.

Although a necessary condition, vegetation stress, and associated atmospheric conditions, either in terms of ignition or propagation of fire, are not a sufficient condition for a wildfire to occur. In the case of 15 October 2017, the number of ignitions was also outstanding and, as shown in Figure 7B, the number of ignitions ranked first, third, and 103 when compared with the same days, months, or the period from 1 April to 31 October respectively. This extremely high number of ignitions on a day characterized by extreme fire weather danger may be partially attributable to the fact that rain was forecasted for 16 October, and most of these ignitions were of human origin and related to agricultural practices.^52^

## Discussion

The 2017 fire season in Portugal was truly exceptional in many ways, namely due to: i) the extraordinarily high value of total burned area at the end of the extended summer (540,000 ha); ii) the burnt area in both months of June and October presenting the highest monthly amounts recorded in 1980–2020 and responsible for 114 fatalities; and iii) the outstanding burned area in October, with more than 200,000 ha occurring in just 24 h and provoking 50 fatalities.

As mentioned before the June episode was triggered by a record-breaking heat wave week in mid-June that affected the entire Iberian Peninsula^38^ along with convection mechanisms responsible for the fast spreading of the wildfires.^60^ Despite the record-breaking heat wave in mid- June and the drought conditions in the region as shown in Figure 3, no NDVI negative anomalies were presented in central and northern Portugal for June (supplemental information Figure S3). It is expectable that the extreme June heatwave accelerated the vegetation stress levels in the Portuguese territory, thus promoting favoring conditions for the October event which is shown to a large extent in the analysis of the SPEI and NDVI that integrate those effects over different time scales.

This work highlights the compound nature of the exceptional fires in Portugal during October 2017 by assessing the different drivers that contributed to it. A particular emphasis is committed to the various components of the meteorological fire danger given by the fire weather index (FWI).

It is worth considering the key drivers of the 15 October compounding event in the framework of the three sides of the traditional “fire triangle” concept (supplemental information Figure S4 and Pyne et al.^70^ and Moritz et al.^71^). In this context, the fuel side is represented by vegetation cover properties, such as biomass amount, connectivity, inflammability and terrain slope that determines the type of combustion that may take place. The oxygen side is determined by weather conditions, namely wind speed and atmospheric instability that determine oxygen availability that feeds the combustion process, as well as air temperature and humidity that regulate the state of fuels. Finally, the heat side is predominantly linked to human activities, either negligent or intentional, which originate fire spots that may spread and intensify according to the type of landscape and the weather.

The exceptional outcome of this event was steered by the combined effect of extended prior meteorological conditions with concurrent fire weather, with FWI reaching an all-time record value on 15 October 2017, as represented in the space framed by coordinates BUI and ISI (Figure 8). Under this perspective, it is clear that the exceptionally high value of FWI on that day is due to the catalytic effect of concurrent exceptionally high value of BUI, reflecting a very high level of vegetation stress with propensity to burn with the also exceptionally high value of ISI, reflecting a propensity of fires to spread at extreme speed. The very high value of BUI resulted from the prolonged drought conditions, whereas the record value of ISI, resulted from exceptionally intense winds associated with the passage of hurricane Ophelia. The resulting record value of FWI translated into previously unseen conditions of meteorological fire danger that, in case of occurrence of ignitions, would likely lead to exceptionally large fire events. The use of FWI as a tool to forecast meteorological fire danger is extensively used in Europe^72^^,^^73^ and also in Portugal.^54^^,^^74^ In particular, the compound event of 15 October 2017 reinforces the need to make use of all components of CFFWIS, in particular, BUI, a slow varying component that reflects the cumulative effects of atmospheric conditions on vegetation stress, and ISI that varies from day to day and is very sensitive to wind speed. On the other hand, assessment of fire danger at short to medium ranges can also take advantage of information provided by ensemble forecast systems,^72^^,^^75^ such as the European Center for Medium-Range Weather Forecasts (ECMWF) or the NCEP operational Global Forecast System (GFS).

The compound nature of droughts and hot extremes have been often associated with disastrous impacts on the ecosystems such as wildfires.^22^^,^^76^^,^^77^ Their occurrence is not regionally limited and did not occur only in Europe^78^^,^^79^ but also in different regions of the globe such as Brazil,^45^ the US^46^ and in Australia.^47^ In all these regions, drought preceding conditions played an important role in intensifying the concurrent heat and wildfire conditions. However, in the case of the October 2017 event in Portugal, the role of a Hurricane was one of the key drivers (and not present in any of the other examples) in the development of the event as previously discussed.

### Limitations of the study

As stated in the discussion section, the compound event approach adopted in this study was in the framework of the classical fire triangle, by looking at the exceptional character of all three sides on October 15, 2017, namely the extreme drought (fuel side), the extreme wind (oxygen side) and the extreme number of ignitions (heat side). The concurring character of the three factors (drought, wind, and ignitions) was analyzed at the daily level and at the scale of the country. However, it is likely that regional and sub-daily factors could also contribute to catalyze the extreme fires, namely atmospheric instability, the daily cycle of meteorological fire danger, and the orientation of the vegetation patches that were affected by the largest fires. For instance, FWI does not incorporate any component that considers the local (or regional) atmospheric instability, a feature that can be relevant in some regions^80^ including Portugal.^60^ This shortcome might be circumvented by using a fire weather index enhanced with atmospheric instability information.^81^ In addition, in this work, the FWI is computed at a daily scale (at 12p.m. local time) and therefore no account was taken of the evolution of the FWI at an hourly timescale during the 15 October, as done in Castellnou et al.^57^ Data for an extended FWI to the hourly scale is already computed^82^ but we believe that it is out of scope in the research here presented. Finally, no analysis was performed on the predominant south-north orientation of some of the vegetation patches most affected by large fires on October 15, in close agreement with the extremely strong southerly winds that blew on that day. The impact of such unusual wind direction in October could be assessed by using a directional FWI, e.g. defined by means of a vector whose direction and magnitude are wind direction and FWI, respectively.

We acknowledge that this work does not follow a standard approach for compound events’ assessments^83^ but follows a storyline structure^84^ instead, aiming to point out the main drivers that contributed the most to the exceptionality of the event.

### Conclusions

Extreme and compound events are expected to be severely exacerbated in a future warmer climate, particularly if a sharp decrease in the emissions of greenhouse gases does not take place in the next two decades.^32^ Compound events are especially threatening to society and the environment, therefore, to provide scientific support to policy making, studies which account for a combination of factors are vital in the context of climate change mitigation and adaptation.

**Figure 9:**
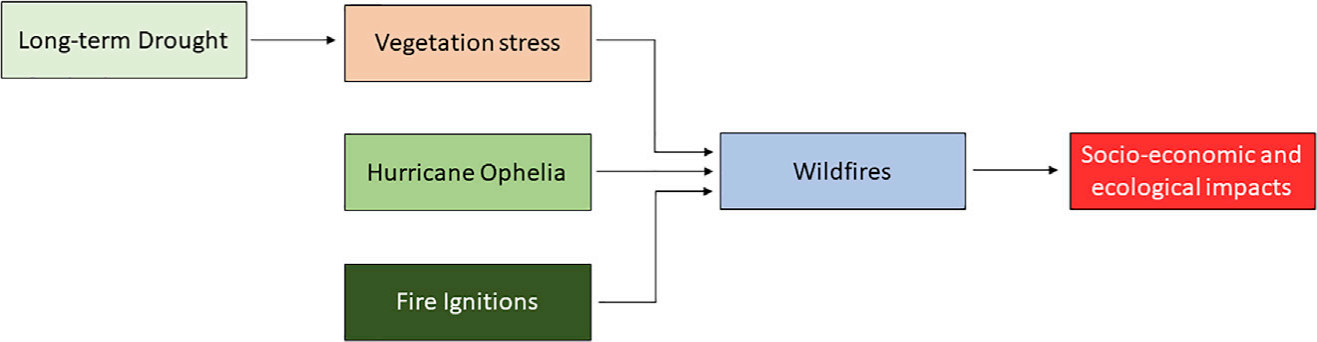
Key drivers of the 15 October compounding event The long-term drought (light green box) that drove the vegetation stress (precondition, in light brown) were amplified on 15 October by two additional drivers (Hurricane Ophelia and ignitions associated with agricultural practices in darker greenish colored boxes). The blue box corresponds to the Hazard (wildfires) and the red one to the socio-economic impacts.

The results are presented in a compound events framework.^22^ These authors refer to compound weather events as a combination of drivers and/or hazards that contribute to societal or environmental risks that potentially cause an impact. This event falls in the preconditioned compound event category from the typology presented in Zscheischler et al.^22^ and the key elements of the 15 October 2017 are summarized in Figure 9. The main climate driver prior to this event was the long-term drought (light green box in Figure 9) that led to preconditioned cumulative hydric stress of vegetation in October 2017 (light brown box). On 15 October 2017, two other major short-term drivers must be considered (darker green boxes in Figure 9), namely: 1) the passage of hurricane Ophelia off the Coast of Portugal responsible for exceptional meteorological conditions (high wind speeds, high temperature, and low relative humidity) and 2) a negligent human driver activity that was responsible for an extremely elevated number of ignitions mostly associated with agricultural practices. This disastrous combination of precondition vegetation stress with the two additional drivers for 15 October 2017 led to uncontrolled wildfires (the hazard, blue box on Figure 9) with extensive fire damage socio-economic and ecological impacts and deaths (red box, Figure 9).

In short, the exceptionality of the meteorological conditions, strong wind speeds, and low relative humidity, associated with the passage of Hurricane Ophelia, along with the preconditioned environment of drought and vegetation hydric stress, were the disastrous background for the higher number of negligent human ignitions becoming uncontrolled wildfires with very dramatic consequences.

## STAR★Methods

### Key resources table

**Table:** 

REAGENT or RESOURCE	SOURCE	IDENTIFIER
**Deposited data**		

ERA5 reanalysis data	ECMWF	https://doi.org/10.24381/cds. adbb2d47
International Best Track Archive for Climate Stewardship Project version 4	NOAA/National Climatic Data Center	https://doi.org/10.1175/2009BAMS2755.1
Monthly gridded climate data	Climatic Research Unit	https://doi.org/10.1038/s41597-020-0453-3
Normalized Difference Vegetation Index	Copernicus Global Land	https://land.copernicus.eu/global/products/ndvi

### Resource availability

#### Lead contact

Further information and requests for resources should be directed to the lead contact, A.M. Ramos (alexandre.ramos@kit.edu).

#### Materials availability

This study did not generate new datasets.

### Method details

#### Data

The fifth generation of the European Center for Medium-Range Weather Forecasts (ECMWF) global reanalysis - ERA5^85^ was used. The ERA5 has hourly data with 137 levels up to 0.01 hPa, which is an improved spatio-temporal resolution, compared to previous reanalysis ERA-Interim. We extracted the following meteorological variables that are available in ERA5: 2m temperature, 2m relative humidity, 10m wind speed, precipitation, and the potential temperature in the lower troposphere, which was computed by using air temperature at pressure levels between 1000hPa and 700 hPa.

In addition, the International Best Track Archive for Climate Stewardship Project version 4 (IBTrACS v4^86^) was used for the Tropical Cyclone (TC) analysis. This dataset contains global information regarding TC activity since 1851, aggregating variables such as geographical location along with maximum wind speed and sea level pressure at 6-hour time steps.

Monthly gridded data from the Climatic Research Unit (CRU) TS4.04 for the 1901–2019 period at 0.5º resolution were extracted and used in this study to analyze drought. The CRU TS4.04 includes cloud cover, diurnal temperature range, frost day frequency, potential evapotranspiration (PET), precipitation, daily mean temperature, monthly average daily maximum and minimum temperature, vapor pressure, and wet-day frequency.^87^ Therefore, it is a dataset suitable for the study of climate variability that has already been successfully used for drought assessment in Iberia.^29^^,^^88^^,^^89^

#### Drought index and Normalized Difference Vegetation Index (NDVI)

The CRU potential evapotranspiration (PET) used is based on the Penman–Monteith equation which was later used to calculate the Standardized Precipitation Evaporation Index (SPEI) for different temporal scales. SPEI is a well-established multi-scalar drought indicator for drought characterization.^89^^,^^90^^,^^91^^,^^92^^,^^93^ Here, SPEI was computed using a log-logistic probability distribution function and the L-moment method for the parameter estimation.^94^ The drought analysis in the present work is constrained to Portugal.

The impact of drought conditions on vegetation health was here assessed by means of the Normalized Difference Vegetation Index (NDVI), as obtained from Copernicus (http://land.copernicus.eu/global/products/NDVI) at 1 km resolution. Copernicus Global Land NDVI product is a 10-day synthesis product derived from Top of Canopy SPOT/VEGETATION or PROBA-V data, which was then converted to monthly means over Portugal for the period ranging from 2000 to 2019. Afterwards, the monthly climatology and respective long-term anomalies (20 years) were computed.

#### The Canadian Forest Fire Index System

The role played by meteorological factors in the occurrence of severe fire episodes is conveniently assessed by means of meteorological fire danger indices that rate the likelihood of a fire event.^95^ One of the most reliable and globally applied fire rating methodologies is the Canadian Forest Fire Weather Index System (CFFWIS) that is based on empirically derived relationships between meteorological variables and the stress of different components of typical fuels that are present in jack pine forests of Canada.^69^^,^^96^ However, since the end of the last century CFFWIS is known to be particularly suitable to rate meteorological fire danger, namely for the ecosystems of Mediterranean Europe.^97^^,^^98^

The system consists of six components that account for the effects of fuel moisture and wind on fire behavior (see Figure). The first three components i.e., the Fine Fuel Moisture Code (FFMC), the Duff Moisture Code (DMC) and the Drought Code (DC) rate the average moisture content of the different layers of the soil.^99^ These components are combined into the Initial Spread Index (ISI), that incorporates wind speed and rates the initial fire spread, and the Build Up Index (BUI) that rates the total amount of fuel available for combustion. Finally, ISI and BUI are combined into the Fire Weather Index (FWI) that rates fire intensity and is suitable as a general index of fire danger. Directly obtained from FWI, the Daily Severity Rating (DSR) is an extension of CFFWIS and rates the difficulty of controlling fires^69^; as a rating of vegetation stress, we will make use of cumulative DSR (DSR_cum_), that, for each day of the year, is simply defined as the accumulated daily values of DSR since April 1 of that year.

**Figure:**
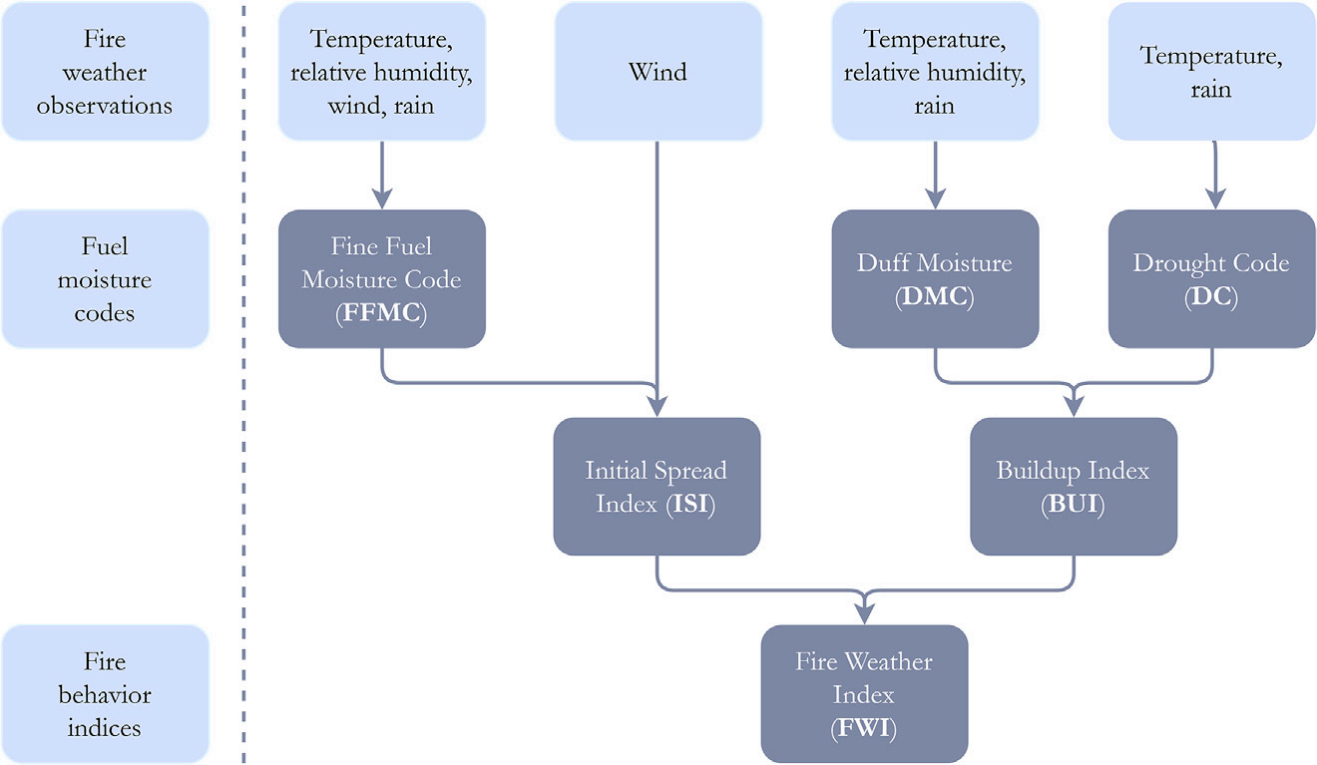
A schematic representation of the Canadian Forest Fire Index System

FWI has been successfully used to define classes of meteorological fire danger over Mediterranean Europe e.g., by fitting a generalized Pareto (GP) model with FWI as a covariate for the scale parameter either to the duration of fire episodes derived from hot spot observations from space^73^ or to the released energy by active fires as derived from satellite observations of fire radiative power.^54^ In turn, DSR has shown to be a suitable parameter to model burnt area variability in Portugal at daily and monthly scales,^100^ to set up wildfire potential outlooks for Portugal by using a statistical model to estimate the probability that the total burned area during summer will exceed a given threshold,^101^ and to improve the predictive capacity of structural wildfire susceptibility and hazard models for Portugal.^102^^,^^103^ The six components of CFFWIS and DSR_cum_ were computed using ERA5 daily values of air temperature, air relative humidity, wind speed and accumulated precipitation at 12 p.m local time.

## Data Availability

• This paper analyzes existing, publicly available data. These accession numbers for the datasets are listed in the key resources table. • Any additional information required to reanalyze the data reported in this paper is available from the lead contact upon request.
