# Supplementary material for: The compound event that triggered the destructive fires of October 2017 in Portugal
Source: iScience. 2023 Feb 4;26(3):106141. doi: 10.1016/j.isci.2023.106141 (PMC10006635; doi:10.1016/j.isci.2023.106141)
Supplement: Document S1. Figures S1–S4 [file mmc1.pdf]

iScience, Volume *26*

Supplemental information

The compound event that triggered

the destructive fires of October 2017 in Portugal

Alexandre M. Ramos, Ana Russo, Carlos C. DaCamara, Silvia Nunes, Pedro Sousa, P.M.M. Soares, Miguel M. Lima, Alexandra Hurduc, and Ricardo M. Trigo

Supplementary material

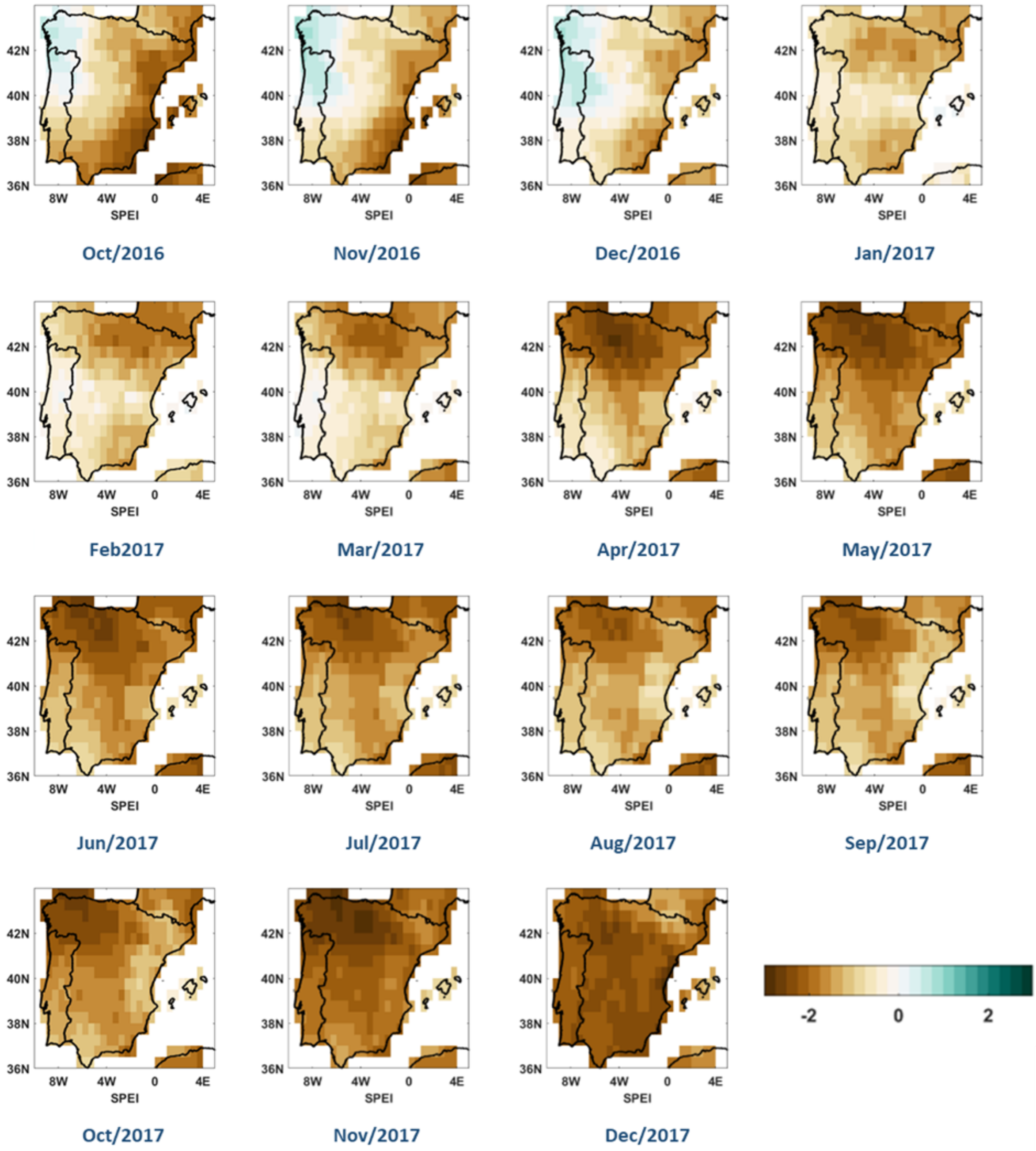

Figure S1. SPEI12 from October 2016 until December 2017 based CRUTS4.04 (1901-2019) on 0.5º resolution, Related to Figure 1.

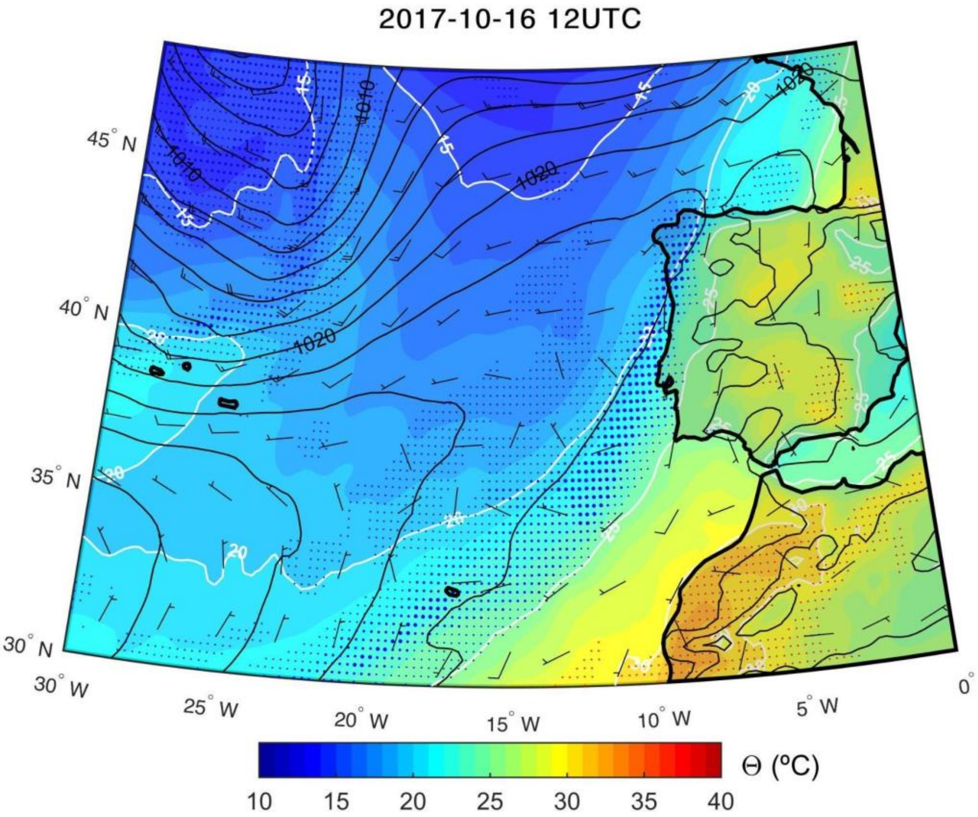

Figure S2. Evolution of the sea level pressure (black contours), potential temperature (colored shading) and relative humidity (blue/red dots) in the lower troposphere (1000-700hPa), as well as surface wind (wind barbs) at 12 UTC 16 October 2017. The increasing size of the blue (red) dots depicts relative humidity values higher than 70%, 80% and 90% (lower than 30%, 20% and 10%), respectively, Related to Figure 5.

.

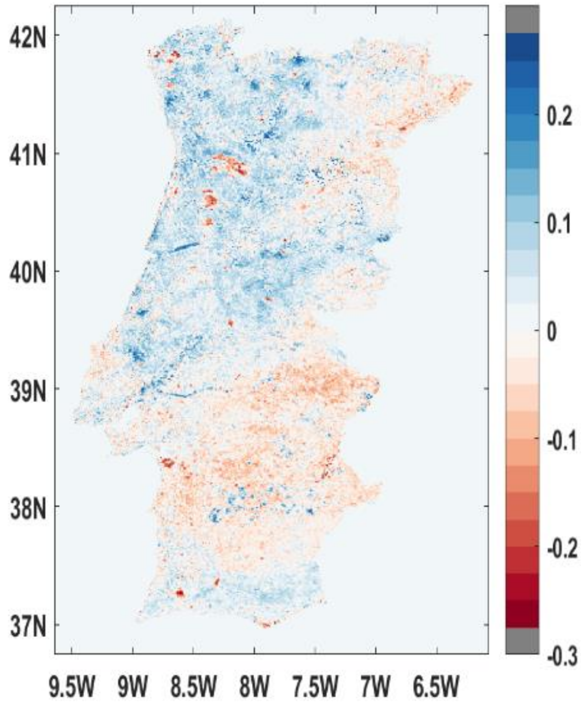

Figure S3. NDVI anomalies for June 2017, Related to Figure 3.

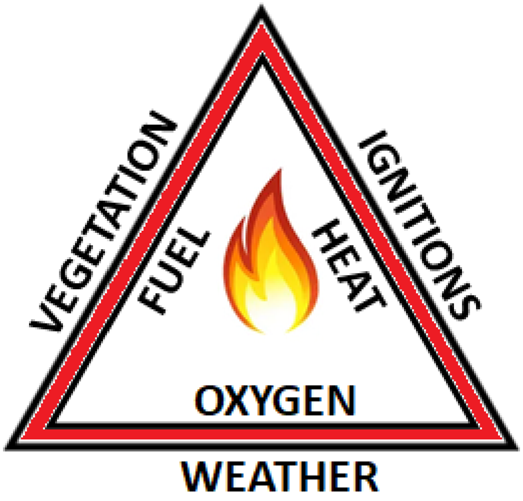

Figure S4. The fire triangle, Related to the Discussion.
